# Monthly Summary

**Published:** 1872-05

**Authors:** 


### MONTHLY SUMMARY.

*Danger from the Vapor of Mercury.*—That metallic mercury gives off vapor in the air at ordinary temperatures has long been admitted. It is even proved that this takes place at a temperature of 32°. A French scientist. M. Merget, referred to in the Catholic *Scientific Press*, has pursued his experiments on this subject with such enthusiasm as that he "expects to be able to prove" that the vapor which mercury constantly sends forth ascends to the height of of 1700 metres, or 1900 feet, at the rate of 200 feet per second. Imagine a surface of quicksilver shooting particles of its substance to a height of the third of a mile with the velocity of a ball fired from a cannon.

Then another class of alleged facts are adduced, and it is asserted that water conveyed in lead pipes carries the metal into the bodies of persons drinking it, where it gradually accumulates and shows its noxious effects—in some cases only after twenty-five or thirty years. As mercury is absorbed and accumulated in the system in the same manner, the logical inference is deduced that it is dangerous to handle mercury, or to inhale the atmosphere exposed to its uncovered surface.

All this sounds well in theory, but is it true in practice? We know that in smelting works and other establishments where metallic vapors are diffused by heat, the most obnoxious consequences may be suffered by persons exposed to the fumes. But are there any facts tending to prove that such results are possible from the presence of mercury in the ordinary temperature of the atmosphere? At the smelting works at New Almaden the workmen were liable to severe poisoning, until pipes were laid to carry the fumes to a distance. Since that time there has been no disease traceable to metallic vapors, and the men whose business it is to bottle the mercury, and who stand constantly over the metal, have never suffered. To say that twenty-five or thirty years is required to test the question of poisoning, is simply to put the whole subject beyond the range of legitimate enquiry. If such latitude be allowed to the argument, we claim that the impregnation of the mercurial atoms, whilst so slight as to produce no noxious results, is a prophylactic against many disorders which would otherwise assail the workmen; and that in the aggregate, the effect is salutary and tends to prolong life. It is not difficult to strain the truths of science so as to extort from them the most absurd deductions. A disposition to do this pervades the medical investigations of our period. It is strikingly exemplified in the exclusive application of the laws of chemistry to the process of life in health and disease, and to the action of medicines. Experiments and experience are labelled "empiricism" and put to shame; and other results than those directly revealed by the senses are cyphered out by theorem. We are getting altogether too theoretical in the practice of medicine, and too regardless of the plain, blunt truths which crop out on the surface, and which are undervalued, because there is no trouble in finding them.—*Pacific Medical and Surgical Journal.*

---

*Purification of Carbolic Acid.*—To remove the unpleasant *odor* usually adhering to the best crystallized acid, Mr. Church, an English chemist, recommends solution in cold water and separation therefrom by common salt. One pound of the crystals is poured into cold water, two gallons or sufficient, after shaking to diesolve all the acid but two or three ounces. This subnatent liquid residue will hold the impurities. The water solution is syphoned off, filtered through fine paper, placed in a tall cylinder, and saturated with common salt. After standing, the—greater part of the—acid rises and may be decanted. It contains about 5 p. ct. water and, of course, remains liquid. The slight odor of pure phenic acid, has a resemblance to oil of geranium; and the addition of this oil has been resorted to, to render carbolic acid pleasant.—*Mich. Univ. Med. Jour.*

---

*On the Effects of Diseased Meat as Food.*—M. Weber having submitted to the Paris Society of Practical Medicine some observations on typhus, and the employment as food, during the siege of Paris, of the meat of cattle that had suffered from aphthous affections, diarrhea, and typhus, M. Vatel expressed his belief that the use of such food was innocuous, provided it was sufficiently cooked. He had, nevertheless, observed the occurrence during that time of a large number of cutaneous diseases and of boils. He was also aware that since 1814, a prejudice has existed against the use of horse flesh, it being considered as giving rise to hæmorrhoids and boils. M. Danet observed that M. Brochin had proved that, except as regards children, no special epidemic was due to aleniation; that typhoid fever, which had prevailed in October, November, and December, 1870, was due to over-crowding; the diseases which prevailed in January and February, 1871, to the influence of cold; and scurvy in March and April to the want of fresh vegetables.—*Cincinnati Medical News.*

